# Supplementary material for: First-line Nivolumab plus FOLFOXIRI/Bevacizumab in advanced RAS/BRAF-mutated colorectal cancer: efficacy, safety and biomarker discovery from the phase II NIVACOR trial
Source: Nat Commun. 2026 Mar 25;17:4478. doi: 10.1038/s41467-026-70620-y (PMC13187018; doi:10.1038/s41467-026-70620-y)
Supplement: Supplementary file 1 — Supplementary Information [file 41467_2026_70620_MOESM1_ESM.pdf]

Supplementary Figures

a

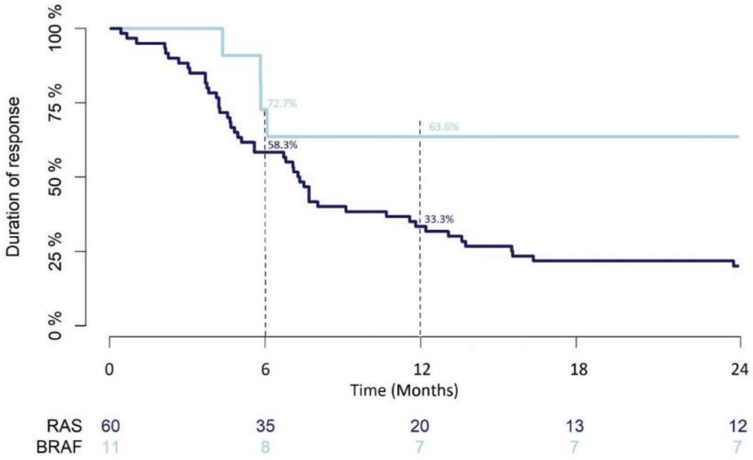

b

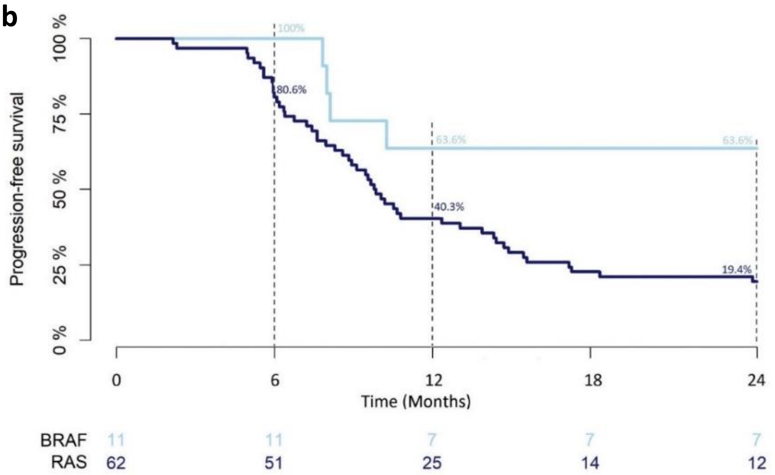

c

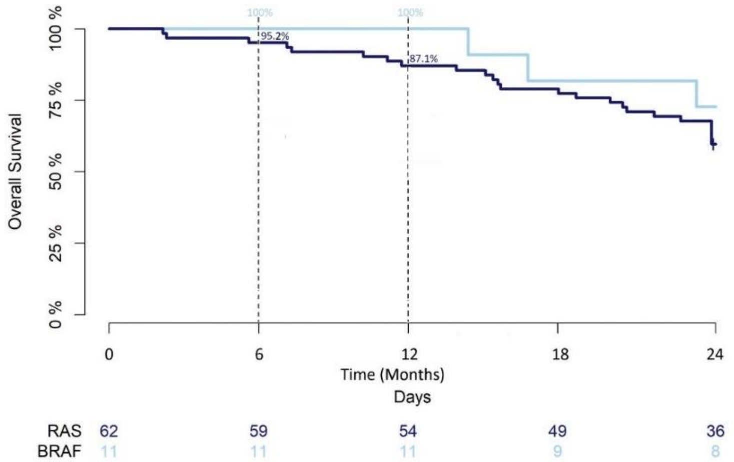

d

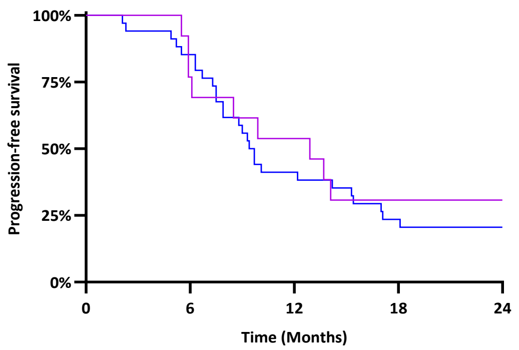

	Number at risk					mPFS (mos)	Log-rank test
RAS G12X	34	29	14	8	7	9.55	Reference
RAS no G12X	13	10	6	4	4	12.9	p=0.607

Supplementary Figure 1: Survival analysis of mCRC patients of the NIVACOR study according to BRAF and RAS mutational status. a-c, Kaplan-Meier curves of duration of response (a), progression-free survival (b) and overall survival (c) of BRAF and RAS mutant patients enrolled in the NIVACOR trial. **d,** Progression-free survival curves of patients carrying RAS G12X mutations compared to patients with other RAS mutations (no G12X), for the subgroup of 47 patients with available information on the type of RAS mutation. The P value was calculated by the two-sided Log-rank (Mantel-Cox) test.

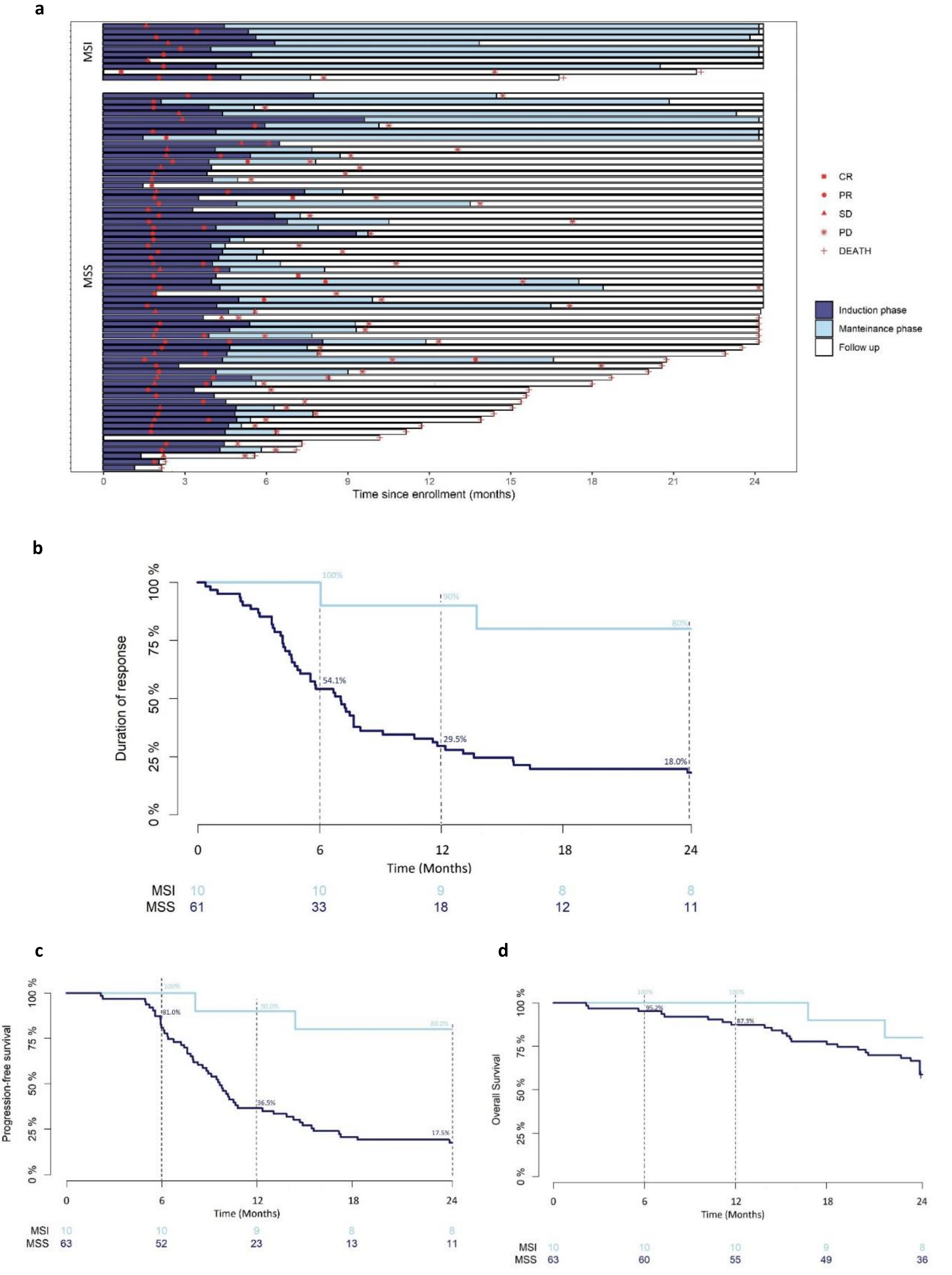
a
MSI
MSS
CR
PR
SD
PD
DEATH
Induction phase
Manteinance phase
Follow up
0
3
6
9
12
15
18
21
24
Time since enrollment (months)
b
Duration of response
100 %
75 %
50 %
25 %
0 %
100%
90%
80%
54.1%
29.5%
18.0%
0
6
12
18
24
Time (Months)
MSI 10 10 9 8 8
MSS 61 33 18 12 11
c
Progression-free survival
100 %
75 %
50 %
25 %
0 %
100%
90.0%
80.0%
81.0%
36.5%
17.5%
0
6
12
18
24
Time (Months)
MSI 10 10 9 8 8
MSS 63 52 23 13 11
d
Overall Survival
100 %
75 %
50 %
25 %
0 %
100%
100%
95.2%
87.3%
0
6
12
18
24
Time (Months)
MSI 10 10 10 9 8
MSS 63 60 55 49 36

Supplementary Figure 2: Tumor response and survival analysis of the mCRC patients of the NIVACOR study according to MSI status. a, Plot of time to response and death in MSI and MSS patients (n=73). Blue bars indicate treated patients in the induction phase with FOLFOXIRI/bevacizumab plus nivolumab; light blue bars indicate treated patients in the maintenance phase with bevacizumab plus nivolumab. **b-d,** Kaplan-Meier curves of duration of response (b), progression-free survival (c) and overall survival (d) of MSI and MSS patients enrolled in the NIVACOR trial.

Abbreviations: CR: complete response; PR: partial response; SD: stable disease; PD: progression disease.

a

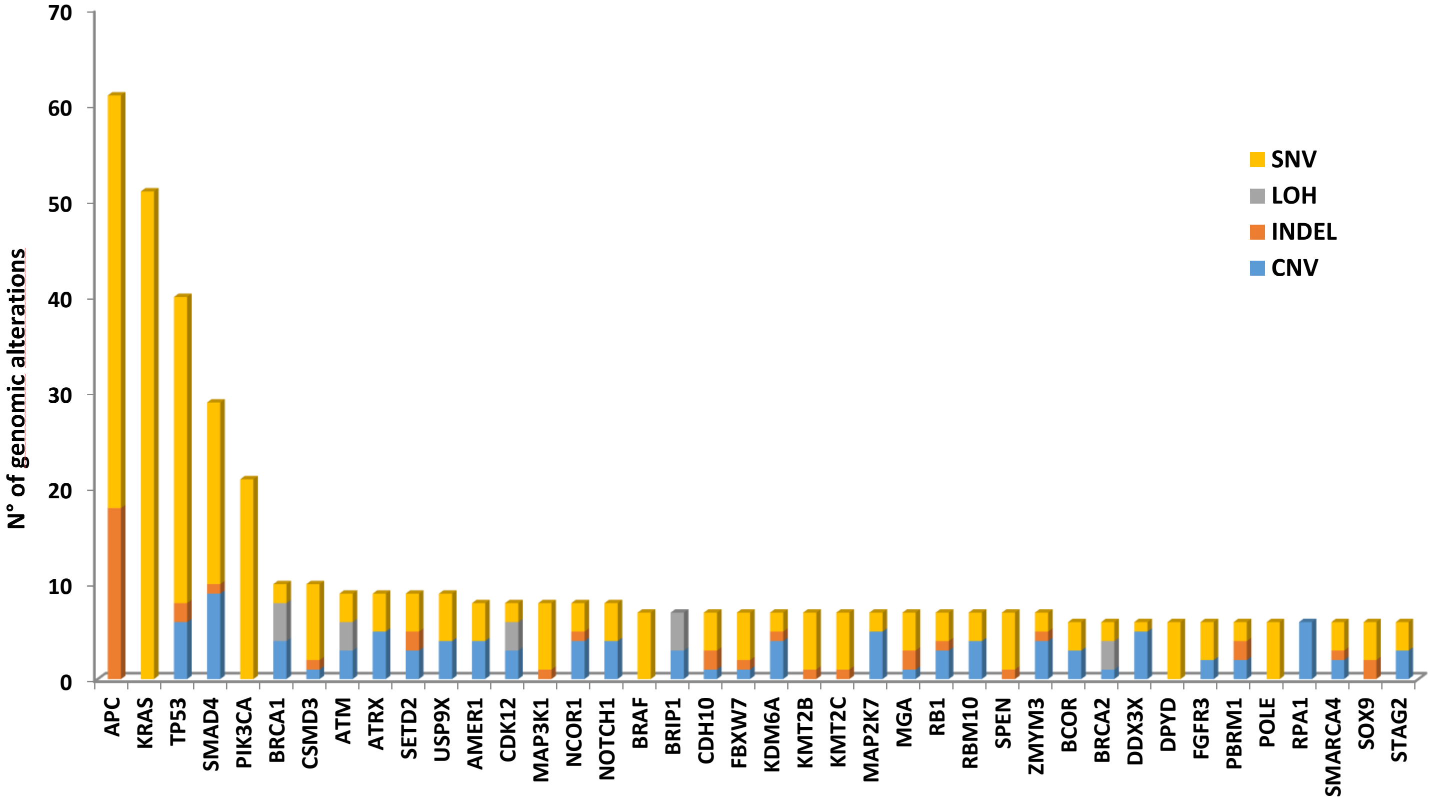

b

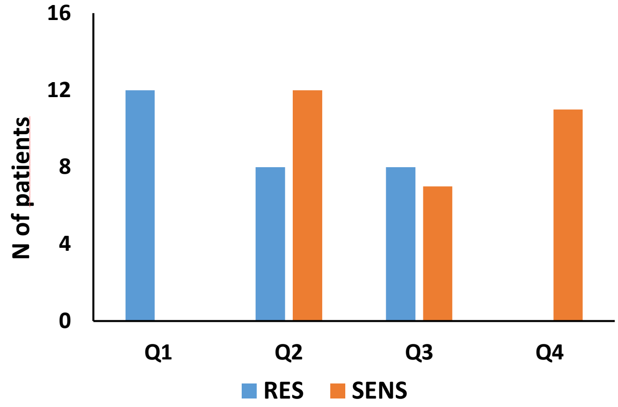

Supplementary Figure 3: Genomic alterations identified by Comprehensive Genomic Profiling and distribution of mCRC patients based on RES and SENS signatures across PFS quartiles. a, Genomic alterations most frequently identified in the 55 patients for which CGP was successfully performed. Only genes with a number of genomic alterations ≥7 are shown. **b**, Distribution of patients with at least one mutated gene in the RES and SENS signatures across the quartiles of PFS (with Q1 including patients below the 25th percentile of PFS and Q4 including patients over the 75th percentile of PFS).

Abbreviations: CGP: Comprehensive Genomic Profiling; PFS: Progression-Free Survival; SNV: Single Nucleotide Variant; LOH: Loss of Heterozygosity; INDEL: Insertion/Deletion; CNV: Copy Number Variation; RES: Resistance signature; SENS: Sensitivity signature; Q: Quartile.

a

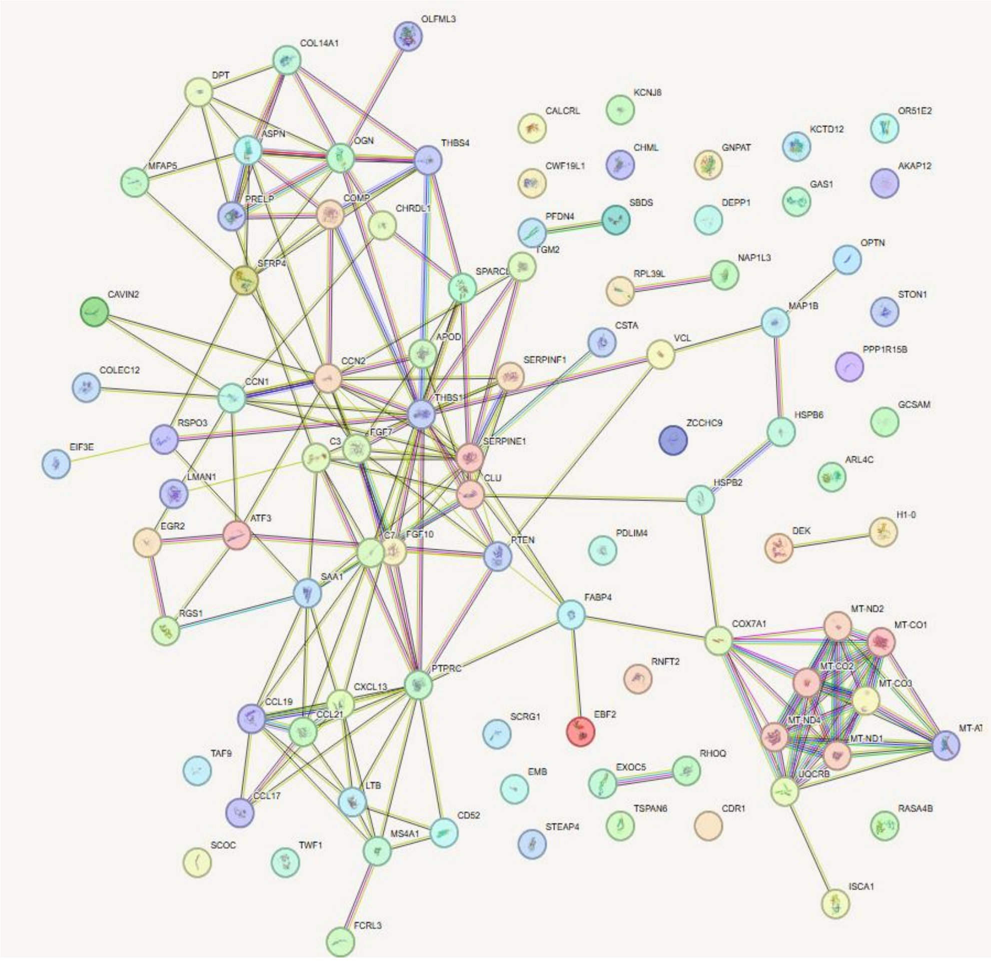

b

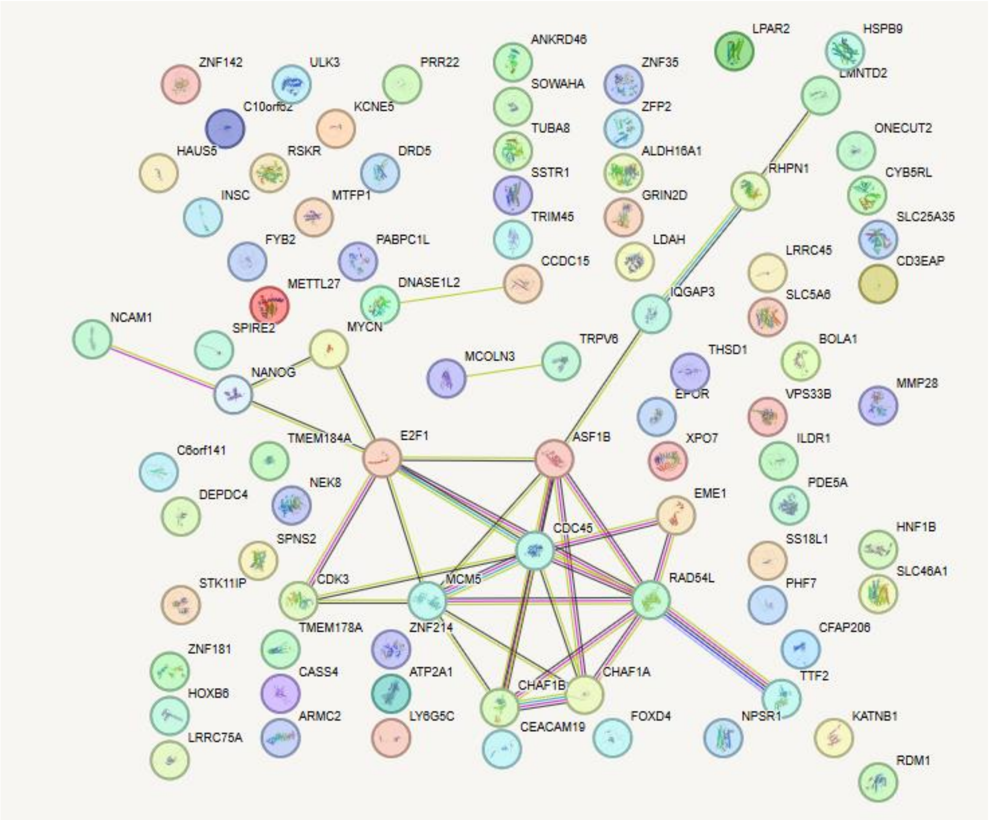

Supplementary Figure 4: Protein-protein interaction analysis of genes differentially expressed between mCRC patients with better and worse prognosis. a-b, Protein-protein interaction networks of upregulated (a) and downregulated (b) differentially expressed genes among patients with PFS above the 75th percentile and patients below the 25th percentile of PFS analyzed by RNAseq (n=48 patients).

Abbreviations: PFS: Progression-Free Survival.

a

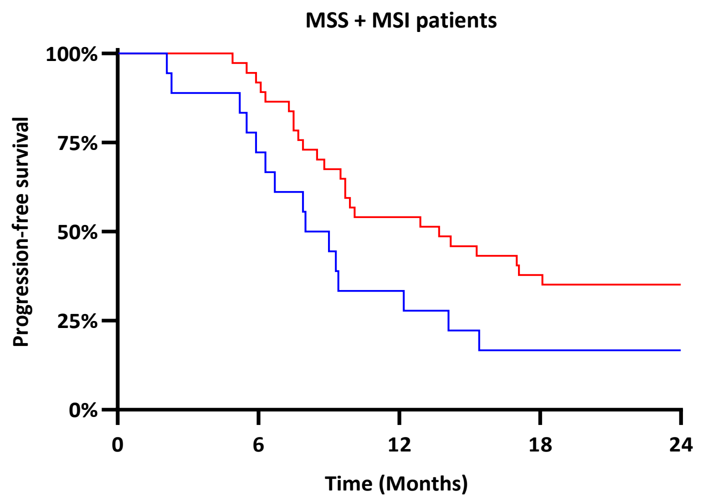

	Number at risk					mPFS (mos)	Log-rank test
PI3K/AKT WT	18	13	6	3	3	8.5	Reference
PI3K/AKT MUT	37	34	20	14	13	13.7	p=0.0431

b

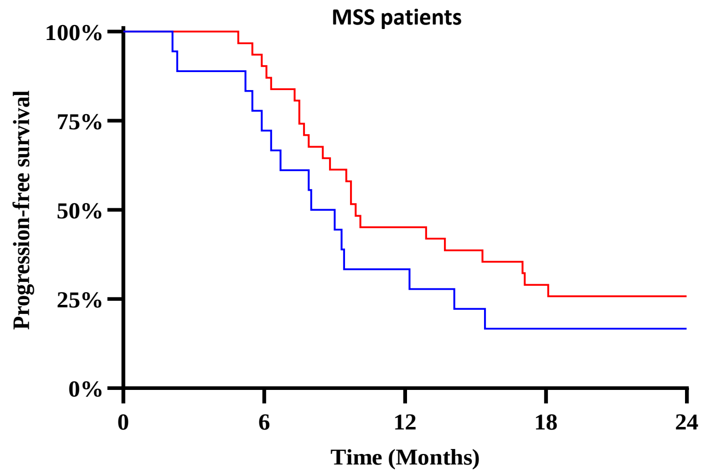

	Number at risk					mPFS (mos)	Log-rank test
PI3K/AKT WT	18	13	6	3	3	8.5	Reference
PI3K/AKT MUT	31	28	14	9	8	9.9	p=0.1955

c

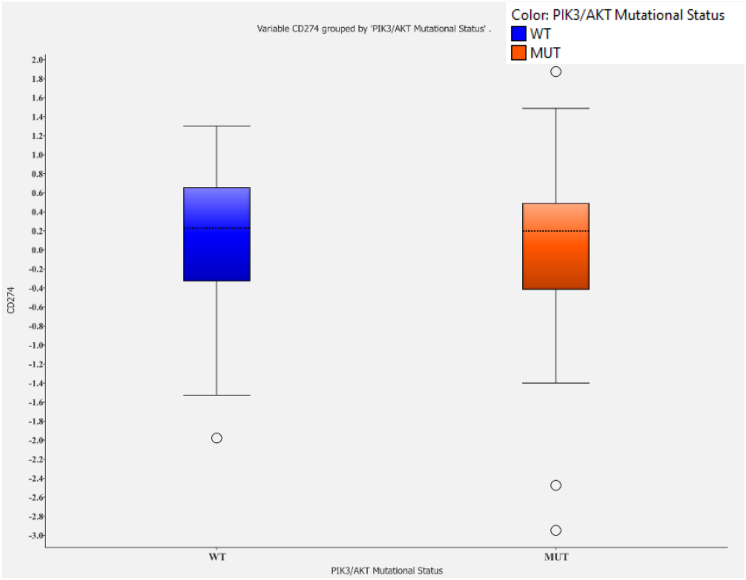

Supplementary Figure 5: Correlation between the presence of genomic alterations in the PIK3/AKT pathway, the PFS and the expression of PD-L1 in mCRC patients. a-b, Kaplan-Meier curves of the PFS for the cohort of 55 (MSS+ MSI) patients analyzed by CGP (a) and the subgroup of 49 MSS (b) patients, according to their mutational status in the PIK3/AKT pathway. P values were calculated by the two-sided Log-rank (Mantel-Cox) test. **c,** Correlation between the levels of expression of PD-L1 (CD274) measured by RNAseq and the mutational status in the PIK3/AKT pathway for the group of patients with available DNA and RNA sequencing data (n=45).

Abbreviations: CGP: Comprehensive Genomic Profiling; PFS: Progression-Free Survival.

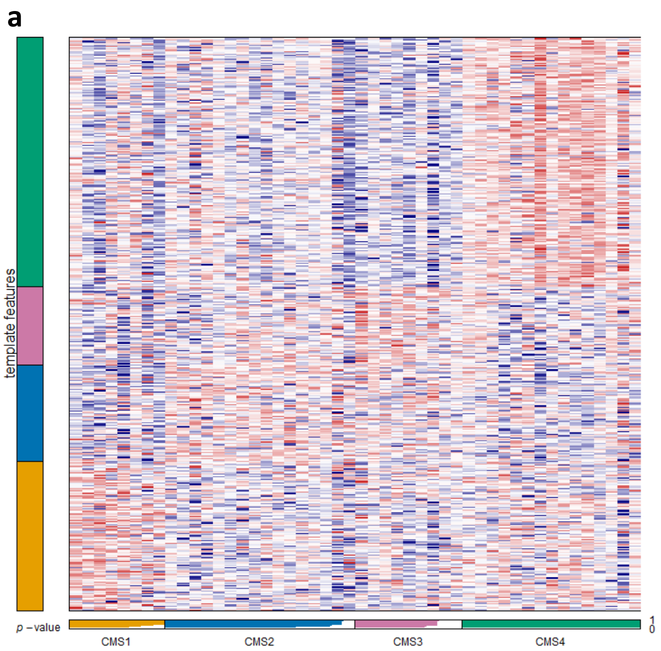

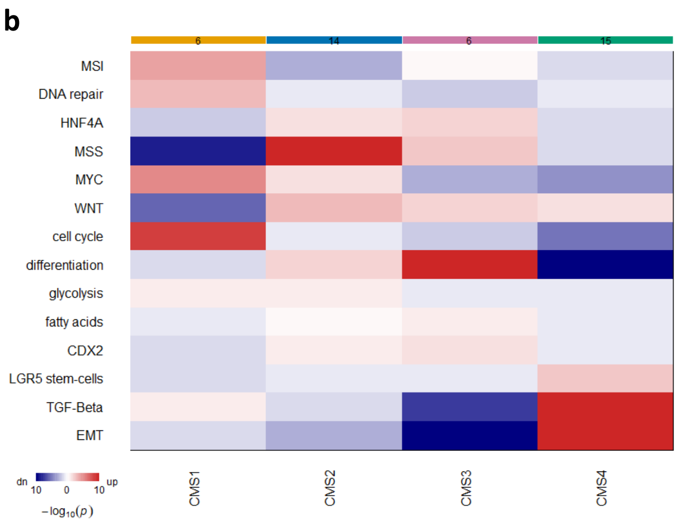

	CMS1	CMS2	CMS3	CMS4	Unclassified
**MSS**	3	14	6	14	5
**MSI**	3	0	0	1	2
**Tot**	**6**	**14**	**6**	**15**	**7**

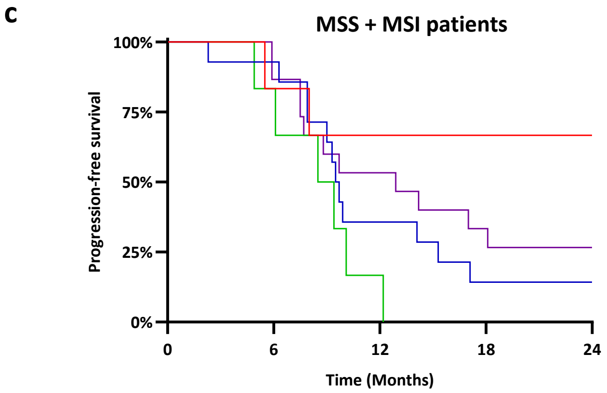

	Number at risk					mPFS (mos)	Log-rank test
CMS1	6	5	4	4	4	NR	Reference
CMS2	14	13	5	2	2	9.6	p=0.086
CMS3	6	5	1	0	0	8.95	p=0.054
CMS4	15	13	8	5	4	12.9	p=0.199

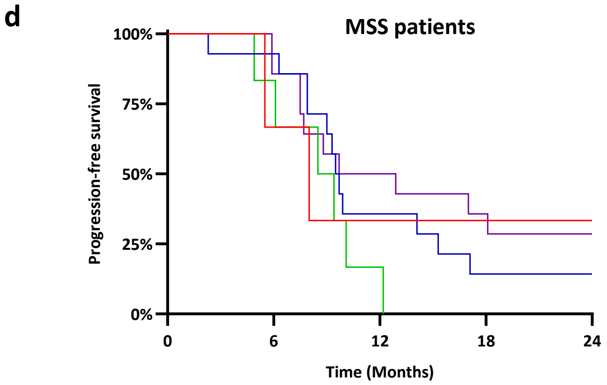

	Number at risk					mPFS (mos)	Log-rank test
CMS1	3	2	1	1	1	8	Reference
CMS2	14	13	5	2	2	9.6	p=0.918
CMS3	6	5	1	0	0	8.95	p=0.640
CMS4	14	12	7	5	4	11.3	p=0.805

Supplementary Figure 6: Consensus molecular subtype classification and correlation with PFS of mCRC patients in the NIVACOR study. a, Heatmap of gene expression profiles for the 48 patients analyzed by RNAseq across the CMS1-4 subtypes. The distribution of MSI and MSS patients among the four CMS subtypes is reported. **b**, Heatmap showing the association between the CMS classification and the relative expression of signaling pathways in mCRC patients. **c-d**, Kaplan-Meier curves of the PFS for the cohort of 41 (MSS+MSI) (c) and the subgroup of 37 MSS (d) patients according to their classification in CMS classes. P values were calculated by the two-sided Log-rank (Mantel-Cox) test.

Abbreviations: CMS: Consensus Molecular Subtype; PFS: Progression-Free Survival.

a

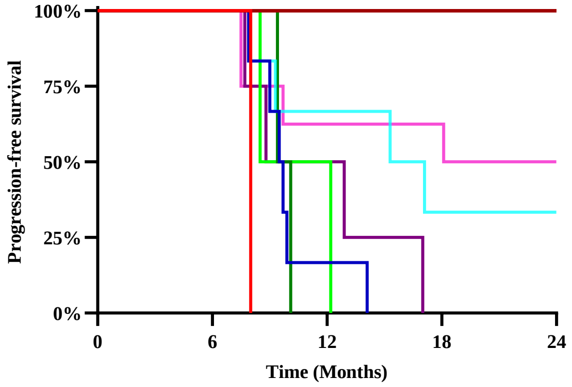

	Number at risk					mPFS (mos)	Log-rank test
CMS1 RES +	1	1	0	0	0	8	Reference
CMS1 RES -	1	1	1	1	1	NR	p=0.317
CMS2 RES +	6	6	1	0	0	9.6	Reference
CMS2 RES -	6	6	4	2	2	16.2	p=0.055
CMS3 RES +	2	2	0	0	0	9.75	Reference
CMS3 RES -	2	2	1	0	0	10.35	p=0.695
CMS4 RES +	4	4	2	0	0	10.85	Reference
CMS4 RES -	8	8	5	5	4	21.05	p=0.108

b

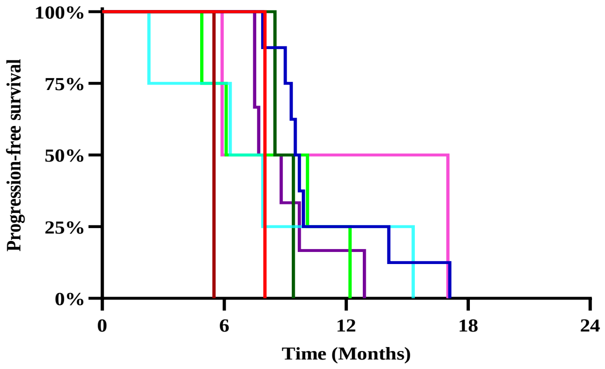

	Number at risk					mPFS (mos)	Log-rank test
CMS1 SENS +	1	1	0	0	0	8	Reference
CMS1 SENS -	1	0	0	0	0	5.5	p=0.317
CMS2 SENS +	8	8	2	0	0	9.6	Reference
CMS2 SENS -	4	3	1	0	0	7.1	p=0.354
CMS3 SENS +	2	2	0	0	0	8.95	Reference
CMS3 SENS -	4	3	1	0	0	8.1	p=0.654
CMS4 SENS +	6	6	1	0	0	8.25	Reference
CMS4 SENS –	2	1	1	0	0	11.45	p=0.457

Supplementary Figure 7: Survival analysis according to CMS classification and RES and SENS signatures after exclusion of patients in the 25th and 75th PFS percentiles. **a-b**, Kaplan-Meier curves of PFS for patients stratified in the CMS classes and for the presence (+) or the absence (–) of genomic alterations in genes belonging to the RES and SENS signatures after exclusion of the 25th PFS percentile subgroup for the analysis of RES signature (a) and of the 75th PFS percentile subgroup for the analysis of SENS signature (b). P values were calculated by the two-sided Log-rank (Mantel-Cox) test.

Abbreviations: CMS: Consensus Molecular Subtype; RES: resistance; SENS: sensitivity; PFS: Progression-Free Survival.

a

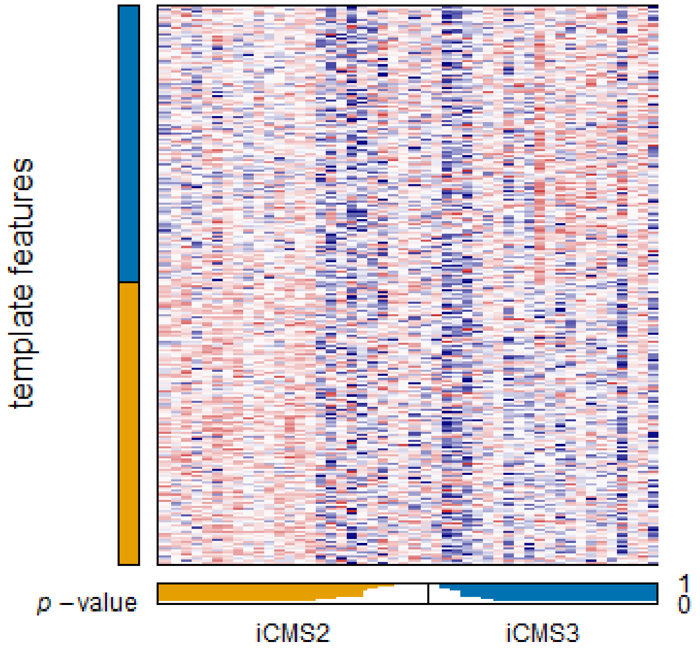

	iCMS2	iCMS3	Unclassified
**MSS**	16	15	11
**MSI**	1	2	3
**Tot**	**17**	**17**	**14**

b

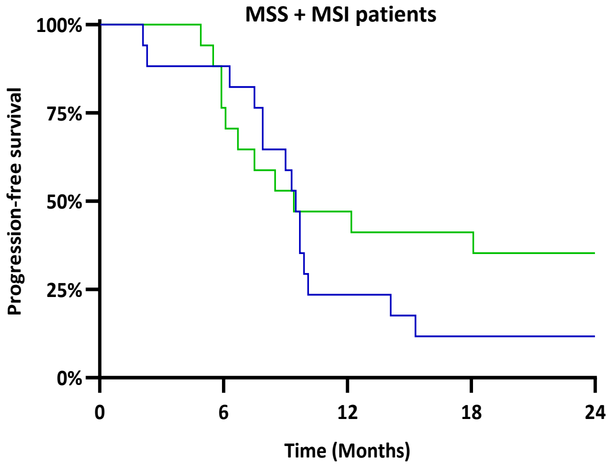

	Number at risk					mPFS (mos)	Log-rank test
iCMS2	17	15	4	2	2	9.5	Reference
iCMS3	17	13	8	7	6	9.4	p=0.313

c

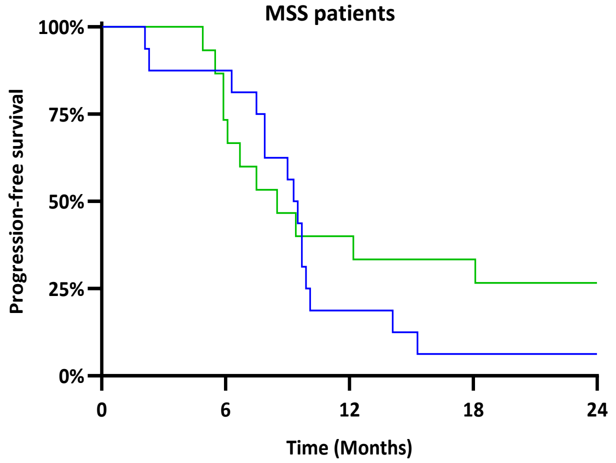

	Number at risk					mPFS (mos)	Log-rank test
iCMS2	16	14	3	1	1	9.4	Reference
iCMS3	15	11	6	5	4	8.5	p=0.452

Supplementary Figure 8: Intrinsic Consensus Molecular Subtype classification and correlation with PFS of mCRC patients in the NIVACOR study. a, Heatmap showing the distribution of 48 patients analyzed by RNAseq in the iCMS2-3 subtypes. **b-c**, Kaplan-Meier curves of PFS for the cohort of 34 (MSS+ MSI) (b) and the subgroup of 31 MSS (c) patients according to their classification in iCMS classes. P values were calculated by the two-sided Log-rank (Mantel-Cox) test.

Abbreviations: iCMS: Intrinsic Consensus Molecular Subtype; PFS: Progression-Free Survival.

a

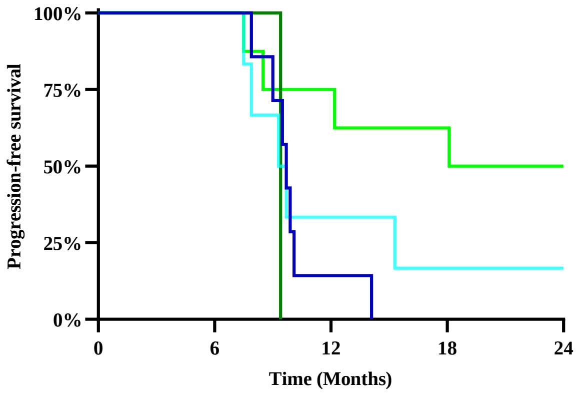

	Number at risk					mPFS (mos)	Log-rank test
iCMS2 RES +	7	7	1	0	0	9.7	Reference
iCMS2 RES -	6	6	2	1	1	9.5	p=0.454
iCMS3 RES +	1	1	0	0	0	9.4	Reference
iCMS3 RES -	8	8	6	5	4	21.05	p=0.068

b

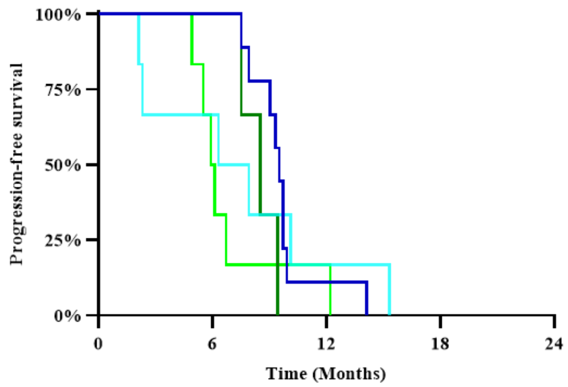

	Number at risk					mPFS (mos)	Log-rank test
iCMS2 SENS +	9	9	1	0	0	9.5	Reference
iCMS2 SENS -	6	4	1	0	0	7.1	p=0.977
iCMS3 SENS +	3	3	0	0	0	8.5	Reference
iCMS3 SENS -	6	3	1	0	0	6	p=0.3965

Supplementary Figure 9: Survival analysis according to iCMS classification and RES and SENS signatures after exclusion of patients in the 25th and 75th PFS percentiles. **a-b**, Kaplan-Meier curves of PFS for patients stratified in the iCMS classes and for the presence (+) or the absence (–) of genomic alterations in genes belonging to the RES and SENS signatures after exclusion of the 25th PFS percentile subgroup for the analysis of RES signature (a) and of the 75th PFS percentile subgroup for the analysis of SENS signature (b). P values were calculated by the two-sided Log-rank (Mantel-Cox) test.

Abbreviations: iCMS: intrinsic Consensus Molecular Subtype; RES: resistance; SENS: sensitivity; PFS: Progression-Free Survival.

Supplementary Tables

Supplementary Table 1. Treatment-related and immune-related Adverse Events in the Overall Population (n=73)

Adverse events	FOLFOXIRI		Bevacizumab		Nivolumab		Overall	
	Any grade n(%)	Grade 3-5 n(%)	Any grade n(%)	Grade 3-5 n(%)	Any grade n(%)	Grade 3-5 n(%)	Any grade n(%)	Grade 3-5 n(%)
**Anemia**	14 (19.1%)	3 (4.1%)	0 (0%)	0 (0%)	0 (0%)	0 (0%)	14 (19.1%)	3 (4.1%)
**Anorexia**	5 (6.8%)	1 (1.4%)	0 (0%)	0 (0%)	3 (4.1%)	0 (0%)	8 (10.9%)	1 (1.4%)
**Bleeding**	2 (2.7%)	0 (0%)	8 (10.9%)	0 (0%)	1 (1.4%)	0 (0%)	11 (15.0%)	0 (0%)
**Central Nervous System Disorders**	1 (1.4%)	0 (0%)	0 (0%)	0 (0%)	1 (1.4%)	0 (0%)	2 (2.7%)	0 (0%)
**Diarrhea**	33 (45.2%)	12 (16.4%)	0 (0%)	0 (0%)	24 (32.8%)	5 (6.8%)	57 (78.0%)	17 (23.2%)
**Electrolyte Abnormalities**	1 (1.4%)	1 (1.4%)	0 (0%)	0 (0%)	2 (2.7%)	0 (0%)	3 (4.1%)	1 (1.4%)
**Fatigue**	26 (35.6%)	3 (4.1%)	0 (0%)	0 (0%)	19 (26.0%)	2 (2.7%)	45 (61.6%)	5 (6.8%)
**Febrile Neutropenia**	3 (4.1%)	3 (4.1%)	0 (0%)	0 (0%)	0 (0%)	0 (0%)	3 (4.1%)	3 (4.1%)
**Fever**	1 (1.36%)	0 (0%)	0 (0%)	0 (0%)	3 (4.10%)	0 (0%)	4 (5.47%)	0 (0%)
**Fistula**	0 (0%)	0 (0%)	0 (0%)	0 (0%)	1 (1.4%)	1 (1.4%)	1 (1.4%)	1 (1.4%)
**Hand and Foot Syndrome**	2 (2.7%)	0 (0%)	0 (0%)	0 (0%)	0 (0%)	0 (0%)	2 (2.7%)	0 (0%)
**Hyperamylasemia and Hyperlipasemia**	0 (0%)	0 (0%)	0 (0%)	0 (0%)	11 (15.0%)	2 (2.7%)	11 (15.0%)	2 (2.7%)
**Hypertension**	0 (0%)	0 (0%)	11 (15.0%)	5 (6.8%)	4 (5.5%)	1 (1.4%)	15 (20.5%)	6 (8.2%)
**Hyperthyroidism**	0 (0%)	0 (0%)	0 (0%)	0 (0%)	5 (6.8%)	0 (0%)	5 (6.8%)	0 (0%)
**Hypertransaminasemia**	5 (6.8%)	0 (0%)	0 (0%)	0 (0%)	3 (4.1%)	1 (1.4%)	8 (10.9%)	1 (1.4%)
**Hypothyroidism**	0 (0%)	0 (0%)	0 (0%)	0 (0%)	12 (16.4%)	0 (0%)	12 (16.4%)	0 (0%)
**Infusional Reaction**	2 (2.7%)	0 (0%)	0 (0%)	0 (0%)	2 (2.7%)	0 (0%)	4 (5.5%)	0 (0%)
**Miastenia Gravis**	0 (0%)	0 (0%)	0 (0%)	0 (0%)	1 (1.4%)	1 (1.4%)	1 (1.4%)	1 (1.4%)
**Mucositis**	17 (23.2%)	3 (4.1%)	0 (0%)	0 (0%)	8 (10.9%)	1 (1.4%)	25 (34.2%)	4 (5.5%)
**Musculoskeletal Pain**	2 (2.7%)	0 (0%)	0 (0%)	0 (0%)	3 (4.1%)	0 (0%)	5 (6.8%)	0 (0%)
**Nausea**	24 (32.8%)	2 (2.7%)	0 (0%)	0 (0%)	12 (16.4%)	0 (0%)	36 (49.3%)	2 (2.7%)
**Neurotoxicity**	30 (41.0%)	2 (2.7%)	1 (1.4%)	0 (0%)	8 (10.9%)	0 (0%)	39 (53.4%)	2 (2.7%)
**Neutropenia**	30 (41.0%)	19 (26.0%)	0 (0%)	0 (0%)	13 (17.8%)	10 (13.6%)	43 (58.9%)	29 (39.7%)
**Ocular Disorders**	0 (0%)	0 (0%)	0 (0%)	0 (0%)	1 (1.4%)	0 (0%)	1 (1.4%)	0 (0%)
**Other Gastrointestinal Disorders**	6 (8.2%)	1 (1.4%)	0 (0%)	0 (0%)	7 (9.6%)	0 (0%)	13 (17.8%)	1 (1.4%)
**Other Genitourinary Disorders**	2 (2.7%)	0 (0%)	0 (0%)	0 (0%)	0 (0%)	0 (0%)	2 (2.7%)	0 (0%)
**Other Hematological Disorders**	7 (9.6%)	1 (1.4%)	0 (0%)	0 (0%)	1 (1.4%)	0 (0%)	8 (10.9%)	1 (1.4%)
**Other Oral Disorders**	6 (8.2%)	1 (1.4%)	0 (0%)	0 (0%)	8 (10.9%)	0 (0%)	14 (19.1%)	1 (1.4%)
**Other Skin Disorders**	1 (1.4%)	0 (0%)	0 (0%)	0 (0%)	4 (5.5%)	0 (0%)	5 (6.8%)	0 (0%)
**Others**	5 (6.8%)	0 (0%)	0 (0%)	0 (0%)	4 (5.5%)	0 (0%)	9 (12.3%)	0 (0%)
**Proteinuria**	0 (0%)	0 (0%)	4 (5.5%)	0 (0%)	0 (0%)	0 (0%)	4 (5.5%)	0 (0%)
**Rash**	2 (2.7%)	1 (1.4%)	0 (0%)	0 (0%)	4 (5.5%)	0 (0%)	6 (8.2%)	1 (1.4%)
**Salivary Gland Infection**	0 (0%)	0 (0%)	0 (0%)	0 (0%)	1 (1.4%)	0 (0%)	1 (1.4%)	0 (0%)
**Thrombocytopenia**	7 (9.6%)	1 (1.4%)	0 (0%)	0 (0%)	0 (0%)	0 (0%)	7 (9.6%)	1 (1.4%)
**Venous Thromboembolism**	0 (0%)	0 (0%)	3 (4.1%)	1 (1.4%)	0 (0%)	0 (0%)	3 (4.1%)	1 (1.4%)
**Vomiting**	9 (12.3%)	0 (0%)	0 (0%)	0 (0%)	11 (15.0%)	0 (0%)	20 (27.3%)	0 (0%)
**Weight Loss**	1 (1.4%)	0 (0%)	0 (0%)	0 (0%)	1 (1.4%)	0 (0%)	2 (2.7%)	0 (0%)

Supplementary Table 2. Clinical and pathological features of patients with available FFPE specimens

Characteristics	All. n = 68
**Age. years. median (range)**	60.5 (32–75 years)
**Sex**	
Male	35 (51.5%)
Female	33 (48.5%)
**MSI Status**	
MSS	58 (85.2%)
MSI	10 (14.8%)
**Somatic Mutation in RAS/BRAF**	
RAS	57 (83.8%)
BRAF	8 (11.7%)
RAS+BRAF	3 (4.5%)
**Site of disease**	
Left colon	30 (44.1%)
Right colon	34 (50.0%)
Transverse colon	4 (5.9%)
**Progression of the Disease**	
Yes	41 (60.3%)
No	27 (39.7%)

Supplementary Table 3. List of genes included in the Resistance signature (n=51)

RESISTANCE SIGNATURE GENES	
PARP3	HRAS
ABL1	KCNH1
ACVR2A	MAP2K4
AGMAT	MGA
APOE	MLH1
ARID1B	MLH3
AXIN2	MRE11
B2M	PALB2
BAP1	PBOV1
BLM	PBRM1
CCND1	PTCH1
CSMD2	PTEN
CYP2C9	RAD51B
DICER1	RB1
DOCK3	RUNDC3B
DSC3	RUNX1
ERAP2	SETD2
ERRFI1	SLCO1B3,SLCO1B3-SLCO1B7
FANCD2	STK11
FANCI	SUFU
FGF3	TAP2
FOXA1	VHL
GIMAP6	XRCC3
GLI3	ZFHX3
GPS2	ZNF831
HDAC9	

Supplementary Table 4. Enrichment analysis for Resistance signature

Index	Name	Adjusted p-value
1	Generic Transcription Pathway	3.257e-8
2	RNA Polymerase II Transcription	9.030e-8
3	Gene Expression (Transcription)	1.460e-7
4	DNA Repair	7.013e-7
5	Resolution of D-loop Structures Through Synthesis-Dependent Strand Annealing (SDSA)	7.134e-7
6	Resolution of D-loop Structures Through Holliday Junction Intermediates	0.000002339
7	Resolution of D-Loop Structures	0.000002339
8	Homologous DNA Pairing and Strand Exchange	0.000005156
9	DNA Double-Strand Break Repair	0.000006480
10	Diseases of DNA Repair	0.000009912

Adjusted p-values were calculated using the Benjamini-Hochberg method for correction for multiple hypotheses testing

Supplementary Table 5. List of genes included in the Sensitivity signature (n=102)

SENSITIVITY SIGNATURE GENES		
CTCF	EIF1AX	PARP4
FAT1	EMSY	PDIA3
FBXW7	EP300	PIK3R1
LATS2	EPHA2	PMS2
MSH3	ERBB2	POLE
RASA1	ERCC2	PPM1D
TAS2R38	ESR2	PRDM1
TCF7L2	ETV1	PTPRT
ABCB1	FGF4	RAD54L
ABRAXAS1	FGF9	RARA
ADAMDEC1	FLT3	RET
ADAMTS12	FLT4	ROS1
AMOT	FNDC7	RPS6KB1
ARAF	FYN	SDHA
ARID2	GATA3	SDHB
ATR	GET1-SH3BGR,SH3BGR	SETBP1
AURKC	GNA13	SIX1
BORCS8-MEF2B,MEF2B	HTR1E	SMAD2
BRAF	IFNB1	SMARCB1
BRIP1	INPP4B	SMC1A
CASP8	KEAP1	SOX9
CASP8AP2	KLF5	SPANXN3
CCND2	KMT2D	SPEN
CDH19	MPP1	STAT3
CDHR4	MPP7	STAT5B
CDK12	MTERF1	TAF1
CHD4	MTOR	TERT
CIC	NBN	TGFBR2
COG1	NF2	TNFAIP3
COL6A5	NOL4	TPP2
CTNNB1	NOTCH2	TSC2
CUL4A	NOTCH3	TTN
DAXX	NSD2	ZNF462
DCAF4L1	PARP1	
DPYD		

Supplementary Table 6. Enrichment analysis for Sensitivity signature

Index	Name	Adjusted p-value
1	Signal Transduction	1.675e-12
2	Generic Transcription Pathway	1.081e-10
3	Diseases of Signal Transduction by Growth Factor Receptors and Second Messengers	3.443e-10
4	RNA Polymerase II Transcription	7.044e-10
5	Disease	1.007e-9
6	Gene Expression (Transcription)	6.575e-9
7	Signaling by Receptor Tyrosine Kinases	2.019e-7
8	PI3K AKT Signaling in Cancer	5.065e-7
9	Developmental Biology	0.000001796
10	Transcriptional Regulation by TP53	0.000003243

Adjusted p-values were calculated using the Benjamini-Hochberg method for correction for multiple hypotheses testing

Supplementary Table 7. Pathway enrichment analysis for up-regulated Differentially Expressed Genes

Index	Name	Adjusted p-value
1	TGF-beta regulation of extracellular matrix	0.0007307
2	FSH regulation of apoptosis	0.01482
3	Binding of chemokines to chemokine receptors	0.02176
4	Oncostatin M	0.02324
5	FGFR2b ligand binding and activation	0.02464
6	BDNF signaling pathway	0.02464
7	AP-1 transcription factor network	0.02464
8	Peptide G-protein coupled receptors	0.02464
9	G alpha (i) signaling events	0.02641
10	Response to elevated platelet cytosolic calcium	0.03361
11	Phagosome	0.03492
12	Alternative complement pathway	0.03492
13	Keratan sulfate degradation	0.03492
14	Integrin signaling pathway	0.03492
15	RhoA signaling pathway	0.03847
16	Lectin-induced complement pathway	0.04865
17	Malaria	0.04865
18	PI3K cascade	0.05786
19	Cells and molecules involved in local acute inflammatory response	0.05786
20	Complement activation, classical pathway	0.05786

Adjusted p-values were calculated using the Benjamini-Hochberg method for correction for multiple hypotheses testing
