# Supplementary material for: First-line Nivolumab plus FOLFOXIRI/Bevacizumab in advanced RAS/BRAF-mutated colorectal cancer: efficacy, safety and biomarker discovery from the phase II NIVACOR trial
Source: Nat Commun. 2026 Mar 25;17:4478. doi: 10.1038/s41467-026-70620-y (PMC13187018; doi:10.1038/s41467-026-70620-y)
Supplement: Supplementary file 4 — Description of Additional Supplementary Files [file 41467_2026_70620_MOESM4_ESM.pdf]

Description of additional supporting information

Supplementary Data 1

Description: Patient characteristics and clinical data
